# Forest Carbon Reserve Calculation and Comprehensive Economic Value Evaluation: A Forest Management Model Based on Both Biomass Expansion Factor Method and Total Forest Value

**DOI:** 10.3390/ijerph192315925

**Published:** 2022-11-29

**Authors:** Jing Zhao, Hui Hu, Jinglei Wang

**Affiliations:** Business College, Southwest University, Chongqing 400715, China

**Keywords:** forest carbon reserves, Gompertz model, carbon fixation efficiency, value evaluation

## Abstract

With the continuous intensification of global climate warming, the carbon cycle has become the focus of global climate change, and the calculation and value evaluation of forest carbon reserves is a key link in promoting the global carbon cycle system. Considering the climatic factors, the biomass expansion factor method (BEF) is used to calculate the forest carbon reserves, selecting the best Gompertz model, adding the time change to the forecast model to predict the growth of forest stock, and the four key indexes of total forest value (TEV) are selected for comprehensive evaluation of forest value. The results show that the carbon fixation efficiency and prediction of forest farms depend largely on the trees, and products can provide more value. Accordingly, it is suggested that broad-leaved trees and younger trees should be planted, and broad-leaved trees should be planted to increase forest stock, increase the vertical distribution of forests to increase carbon reserves, and make trees into wood products with longer retention time to achieve higher total forest value.

## 1. Introduction

The carbon cycle is the most important cycle in the Earth’s ecosystem. It controls the main material cycle of the terrestrial ecosystem and affects the environment on which human beings live [1]. However, human activities have caused a huge impact on the carbon cycle since the industrial revolution; due to a large amount of fossil fuels burning and unreasonable use of land, human activities emitted a lot of carbon into the atmosphere. The problems caused by it have concerned all countries in the world.

Forest ecosystems acting as an absorptive CO_2_ and release O_2_ carbon sink play a crucial role in the carbon cycle. Previously, relevant scholars at home and abroad have put forward CBM-CFS3, Bern2.5 CC, and other research models [2,3]. Compared with before, only relying on reducing greenhouse gas emissions, carbon capture and storage have gradually received attention. There are two main effective means, namely industrial carbon sequestration and biological carbon sequestration. Biocarbon sequestration is the amount of wood carbon sequestration to infer the amount of carbon sequestration of forests [4], and forest carbon sequestration is inferred by calculating the amount of wood carbon sequestration, but such means have some limitations. At present, there are three main methods. The first one is the average biomass method, which obtains the diameter order material product growth amount through the material product growth rate of the diameter order [5]. Although this method is relatively small, the field workload is large, and the sampling system is disturbed and the scale transformation has certain errors; remote sensing technology and isotope tracing technology are also commonly used. Although these two methods are relatively simple, they still have large errors. In addition, including geological storage, marine storage, ore carbonization, and industrial carbonization, the industrial carbon storage method is receiving more and more attention, although the results of carbon storage calculated by these methods are relatively accurate but, as they are more expensive, with large investment, no carbon dioxide storage in living plants, and without young forest regeneration and sealed carbon combination, have certain limitations.

In the existing studies, the specific calculation methods of forest carbon sequestration are relatively diversified. The more common methods include forest resource inventory method, model simulation, remote sensing monitoring, carbon flux monitoring, and atmospheric inversion method. It is a common method for many researchers to calculate the carbon reserves and remote sensing parameter variables of the sample plots by using the survey data and remote sensing images and to build a multi regression model to analyze the regional forest carbon reserves [6]. Some also estimated net primary productivity and carbon fixation potential by process-based remote sensing model (CASA model) [7]. Some scholars estimate terrestrial carbon density (ACD) by combining airborne LiDAR canopy height measurements with Planet Dove satellite imagery [8]. A method for estimating carbon stocks in Japanese cedar forests has also been described that uses a high-resolution, helicopter-borne three-dimensional (3D) scanning lidar system to measure the three-dimensional canopy structure of each tree in a forest [9]. There are also studies that first estimated regional accumulation of wood volume in fir and cypress over time, expressed biomass allocation fraction as the age function of two forest types, calculated conversion factors over time, and estimated carbon reserves of all tree species using forest age statistics [10]. Some scholars used a development regression method, with SAR integrated dual sensors to estimate forest carbon reserves [11]. A correlation method based on bioclimatic envelope models and data from inventoried forest plots has been used to calculate forest carbon stocks [12]. Other scholars used anisotropic growth models to estimate the carbon stock of forests [13,14].

In addition to the above methods, it has become a relatively common method to estimate forest biomass by using the biomass expansion factor method (BEF), and determining how to further improve the accuracy of estimation has become a popular research topic among many scholars. Some scholars changed the fixed biomass average conversion factor into provincial region and age group, using the biomass regression model method, which improves the accuracy of taking the biomass and stock ratio as the constant calculation [15]. There is also direct use of BEF for carbon stock estimation [16]. Differences between biomass estimates obtained using biomass equations and BEFs have also been evaluated [17]. There are studies that have proposed equations for improving forest biomass and carbon reserves by showing the dependence of tree size and age on BEF and R [18]; several studies have presented measurements that represent the largest available dataset tree to estimate BEF and R [19]. Other scholars have combined the BEF with the biomass allometric equation to assess the forest carbon reserves [20,21]. In addition, biomass regression equation and BEF are combined to evaluate forest carbon reserves [22,23].

While making more and more accurate determination of forest carbon reserves, determining how to better improve the value of forest has also gradually been paid attention by experts and scholars. However, there are few studies evaluating forest value alone, and most forest evaluation activities use prescribed preference methods, such as market estimation method, distributed measurement method, hypothesis development method, shadow price method, and consumer willingness to estimate the value of forest [24,25,26]. Some academics use the value functions approach in modeling stakeholder values in regional forest planning [27]. Through the analysis of social benefits and social costs (or B/C analysis), the forest management scheme can be evaluated [28]. Some scholars proposed a nonmarket-oriented explicit preference technique to identify all possible forest values and trigger preferences for different forest values [29]. More research on forest value is carried out based on the calculation of carbon reserves. Based on the estimated forest biomass results, the biomass: carbon reserves conversion coefficient method is used to estimate carbon reserves, and the carbon tax law is used to evaluate the value of forest carbon reserves. Other scholars measured carbon sequestration by the gain: loss method using international forestry data provided by the International Panel on Climate Change (IPCC), from which the TEV is calculated [30].

To summarize, there are many methods to calculate forest carbon reserves, among which using BEF to estimate carbon reserves has become a popular method. Many scholars continuously improve the accuracy of BEF through a series of models and index optimization, but the comprehensiveness and integrity still need to be improved.

In order to alleviate the global warming caused by greenhouse gases, it is not enough to rely on nature’s own regulation, but it is necessary to explore long-term effective methods from economic and technological aspects, as well as to make reasonable suggestions on how forests can achieve the maximum carbon sequestration, find ways to achieve the maximum carbon sequestration in forests, and ultimately promote the carbon cycle. In this study, forest carbon storage was quantified by biomass expansion factor method (BEF), and the influence of forest species on forest stock was analyzed by fitting forest growth model, and the indicators of total economic value model (TEV) were used to evaluate forest value to make the best suggestions for forest managers.

## 2. Materials and Methods

### 2.1. Study Design

Based on existing studies, this paper establishes a carbon sequestration model to determine the CO_2_ in which forests can persist over time measure. Carbon reserves include the sum of changes in the carbon reserves in a forest minus the increase in greenhouse gas emissions within them.

By constructing the biomass expansion factor model and the carbon fixation rate model of wood products, the carbon dioxide retention of the whole forest and its products over time was determined, as well as how to change the management mode to simply improve the carbon fixation efficiency with only the area and the stock of different trees. In addition to forest biomass, a forest carbon bank also includes shrub biomass, litter biomass, dead biomass, and carbon reserves of easily overlooked but critical production products. In this paper, we use the biomass expansion factor method to quantify the carbon stock of four forests using the forest area and tree stock of different tree species.

Moreover, this paper uses a model fitting with a timeline strategy to predict forest stock growth after 100 years, iterating on different forest species growth models over 100 years and adding temporal variables to assess forest stock after 100 years. In this paper, different tree growth data are compared and fitted with five biological models and, through horizontal comparison, the Gompertz model of the optimal growth curve of tree volume with age is obtained. Based on the calculation of forest carbon reserves, the forest asset resource value evaluation model is constructed based on the TEV model, and four key evaluation indicators are selected to evaluate the various values of the forest, and, finally, the appropriate management plan is determined. We use existing forest data to assess forest values and compare quantitative value weights to make management plans for forest managers that best fit carbon sequestration.

The model in this paper is more carefully modeled than other previous models, the optimized model is more rigorous, and a new method of biological factor analysis is to use quantitative extension factors, which are more scientific and more accurately describe forest carbon reserves under specific conditions. In addition, the model takes into account the useful life of forest products when assessing forest products, making the estimates of forest biomass and carbon sink more accurate.

### 2.2. Hypotheses and Arguments

In order to improve the accuracy of the research findings, the constraints of the realistic environment need to be sorted out. This paper makes the following assumptions to avoid the interference of certain factors on the research process and conclusions. Since the focus and main contribution of this paper is for the combined innovation of BEF and growth factor method and the value evaluation, the uncertainty of the hypothetical conditions does not affect the credibility of the conclusions of this study.

**Hypothesis 1.**  *The model builds that no forest fires occur under natural conditions. The main natural cause of forest fires is climate change, and the key trigger is a sustained or sharp rise in temperature, which occurs in summer. The forests selected in this paper are located in coastal areas, and the possibility of fire is unlikely. Therefore, in the process of model construction, extreme dry weather and human behavior are excluded, assuming that forest fire will not occur under natural conditions [31].*

**Hypothesis 2.**  *CO_2_ content does not change when logging is carried out by logging machines, but different logging methods and intensity of logging can affect soil carbon stocks and carbon sequestration. It affects forest CO_2_ emission rate and carbon sequestration by affecting soil temperature, soil water content, and soil nitrogen content after logging, rather than directly making the content of CO_2_ changes. Therefore, this paper assumes that logging machinery does not cause changes in the content of CO_2_.*

**Hypothesis 3.**  *The carbon reserves in unit woodland are certain. Some scholars have used remote sensing observation data to analyze the relationship between regional net primary productivity and climate and have found that the change in soil utilization has relatively little impact on the overall carbon cycle [32]. In this paper, we consider that the major difference between restricted and unrestricted areas is the difference in soil carbon content. By testing whether disturbance intensity affects carbon stocks over time, we first remove plots that do not belong to forest or savanna biomes or have no recorded biology. Then, the model was adjusted for standard deviation by constructing ensemble models, performing predictions and performing uncertainty analysis, and sampling all environmental covariates. The results show that the carbon reserves within the unit woodland are certain and are applicable in any area [33].*

**Hypothesis 4.**  *Forests do not produce a large-scale carbon loss in a short period of time. To assess the underlying drivers of changes in carbon accumulation rates, this paper investigated that carbon loss did not show large differences in biomes over a short period of time. For litter and coarse wood chips, the carbon reserves either showed a nonlinear decline from the initial conditions or did not produce a large-scale carbon loss in a short time. Considering the age of trees to study the change in biocommunity, the depth was used as a predictor and the carbon loss did not change greatly.*

### 2.3. Data Source

In this study, four forest farms, located in Shaoguan city, Guangdong Province, China, were selected. The data are from the certified emission reduction record table of Wengyuan County, Shaoguan City, released by the Department of Ecology and Environment of Guangdong Province. The data used in the study were all from authoritative websites or the literature, which ensured the accuracy and rigor of the data, overcame the difficulties of data collection, and, to a certain extent, ensured the accuracy of the carbon stock assessment.

Wengyuan County, Shaoguan City, Guangdong Province, China, is located in southern China; east longitude 113°39′2″ to 114°18′5″, north latitude 24°07′30″ to 24°37′15″. It is 66.5 km long from east to west and 55 km wide from north to south, with a total area of 2175 square kilometers. It belongs to the middle subtropical monsoon climate. The annual average temperature is 20.6 °C. The annual average rainfall is 1693.9 mm and the annual average sunshine is 1586.2 h, with more sunshine. Spring is from March to April, summer is from May to September, autumn is from October to November, winter is from December to February of the next year, and summer lasts for five months. The average temperature of more than 90% of the years in winter is above 10 °C, which is suitable for crop growth.

The natural land (the soil under natural vegetation that has not been reclaimed and utilized by humans) [34] of Wengyuan County is 191,283 hectares, accounting for 88.7% of the total land area of the county. The soil mainly includes yellow earths, rhodic, purplish soil, black calcareous soil, red calcareous soil, and paddy soils. Mountain vegetation in Wengyuan County belongs to the subtropical evergreen season wind and rain belt, and the vegetation presents a diversity, mainly including herbaceous vegetation, coniferous and broad-leaved mixed forest, and sparse forest and grass slope.

### 2.4. Research Methods

#### 2.4.1. Carbon Sequestration Model Based on BEF

Carbon reserves include the sum of changes in the carbon reserves in a forest minus the increase in greenhouse gas emissions within it. In most forests, the carbon reservoir mainly includes forest biomass carbon reserves, forest shrub biomass carbon reserves in the forest, dead biomass carbon reserves in the forest, biomass carbon reserves of dead litter in the forest, and carbon reserves of wood products harvested in the forest. Under ideal conditions, unless forest fires and other unnatural projects occur, the forest itself would not produce additional CO_2_ and the amount of CO_2_ that can be sequestered by the forest and its products over time can be deduced by simply calculating the annual change in the forest carbon pool.

(1) $$\Delta C_{P,t}=\Delta C_{TREE,t}+\Delta C_{SHRUB,t}+\Delta C_{DW,t}+\Delta C_{LI,t}+\Delta C_{HWP,t}$$

$\Delta C_{P,t}$ is the change in carbon reserves at year *t*; $\Delta C_{TREE,t}$ is the biomass carbon reserves in the forest at year *t*; $\Delta C_{SHRUB,t}$ is the change in carbon stock of shrub biomass at year *t*; $\Delta C_{DW,t}$ is the change in carbon stock of dead biomass at year *t*. $\Delta C_{LI,t}$ is the change in carbon stock of deadfall biomass at year *t*. $\Delta C_{HWP,t}$ is the change in carbon stock of harvested wood products at year *t*.

(1) Forest tree biomass and carbon reserves in the forest

(2) $$C_{TREE,t}=\frac{44}{12}*\underset{j=1}{\sum }\left(B_{TREE,j,t}*CF_{TREE,j}\right)$$

Forest carbon stock refers to the sum of the product of the biomass accumulated by all the different tree species themselves and the carbon rate in their biomass over a certain period of time and converts the resulting C into a molecular weight ratio of ${\mathrm{CO}}_{2}$. $CF_{TREE,j}$ is the carbon content rate in the biomass of tree species *j*. $B_{TREE,j,t}$ is the biomass of tree species *j* at year *t*. Where the change in the value of $CF_{TREE,j}$ is weak and can be measured once, $B_{TREE,j,t}$ can be calculated using the biomass expansion factor method. The specific calculation method is as follows:

(3) $$B_{TREE,j,t}=V_{TREE,j,t}*D_{TREE,j}*BEF_{TREE,j}*\left(1+R_{TREE,j}\right)*N_{TREE,j,t}*A_{t}$$

The biomass expansion factor method calculates stand biomass in three steps: in the first step, the tree height (H) or diameter at breast height (DBH) is converted into tree trunk volume by using the wood volume formula or by checking the wood volume table; in the second step, stand trunk wood volume is converted to stand aboveground biomass using biomass expansion factor (BEF) and basic wood density (D); in the third step, aboveground biomass is converted to stand biomass by the ratio of belowground biomass/aboveground biomass (R). $V_{TREE,j,t}$ is the accumulation of tree species *j* harvested by the project in year *t*. $D_{TREE,j}$ is the basic wood density of tree species *j*. $BEF_{TREE,j}$ is a biomass expansion factor of tree species *j* for converting trunk wood volume into aboveground biomass of the stand. $R_{TREE,j}$ is the ratio of belowground biomass/aboveground biomass of tree species *j*. $N_{TREE,j,t}$ is the number of strains of tree species *j* in the *i*-th baseline carbon layer and $A_{t}$ is the area of the forest at year *t*.

(2) Biomass carbon reserves of shrubs in the forest

(4) $$C_{SHRUB,t}=\frac{44}{12}*CF_{S}*\left(1+R_{S}\right)*A_{t}*B_{SHRUB,t}$$

The calculation of shrub biomass is based on the same idea as that of forest biomass. $CF_{S}$ is the carbon content rate in shrub biomass and $R_{S}$ is the ratio of belowground biomass to aboveground biomass of the shrub. Usually, the species of forest shrubs is relatively fixed, the biomass and carbon content will not change greatly, and you can directly use the default value instead. The default value of $CF_{S}$ is 0.47 and the default value of $R_{S}$ is 0.40. $B_{SHRUB,t}$ is the average aboveground biomass of shrubs per hectare in the carbon layer at year *t*. Calculations based on the actual:

Shrub cover < 5%, average shrub biomass per hectare can be considered as 0;

Shrub cover ≥ 5% was estimated according to the following formula:

(5) $$B_{SHRUB,t}=BDR_{SF}*B_{FOREST}*CC_{SHRUB,t}$$

$BDR_{SF}$ is the ratio of the average shrub biomass per hectare at 1.0 to the average forest biomass per hectare of the project implementation area, $B_{FOREST}$ is the regional average aboveground biomass per hectare of forest, and $CC_{SHRUB,t}$ is the shrub cover of the carbon layer at year *t*, expressed as a decimal.

(3) Biomass carbon reserves of dead wood in the forest

(6) $$C_{DW,t}=C_{TREE,t}*DF_{DW}$$

Dead biomass carbon stocks were calculated using the default factor method. $DF_{DW}$ is the ratio of dead wood carbon reserves and living wood biomass carbon reserves in the forest. Dead biomass of different tree species is related to climate and environmental impact; among them, China has the most extensive and representative climate. According to the national forest resources inventory stand stock and dead stock in China 1994–1998 and 1999–2003, the default values in Table 1 were adopted:

(4) Biomass carbon reserves of dead litter in the forest

(7) $$C_{LI,t}=C_{TREE,t}*DF_{LI}$$

In forests, because the inventory of dead objects is very inconvenient, the following default equations are used in most cases. The specific reference data of parameters a and b are obtained from the United Nations Intergovernmental Panel on Climate Change, as shown in Table 2. $DF_{LI}$ is the ratio of dead litter carbon reserves and living wood biomass carbon reserves in the forest. If there are many wood species that are not easy to clear, the IPCC recommended $DF_{LI}$ = 4% can be used.

(8) $$DF_{LI}=a\cdot e^{b\cdot B_{{TREE}_{-}AG}}$$

(5) Changes in the carbon reserves of the harvested wood products

If deforestation occurs in a forest, the long-term change in carbon stocks in wood products is equal to the carbon in wood products that are still in use at the end of the forest period or 30 years after the production of the product and that go to landfill. Other parts are considered to be emitted immediately during the production of wood products. The change in carbon reserves of wood products is estimated by the following methods.

First, the subsequent usage of the produced products is calculated to determine the time from IPCC to the replacement of the management plan (calculated here based on the growth cycle of trees from planting to logging). The following calculation method is adopted:

(9) $$OF_{ty}=e^{\left(-\ln\left(2\right)*WT/LT_{ty}\right)}$$

In the actual production of wood products, the wood density of the species is the key to lumber and, the higher the wood density, the less logs will be consumed. $OF_{ty}$ is the proportion of wood products still used and entered in the landfill 30 years after their production, so the density of wood needs to be considered when calculating the consumption of products. At the same time, most products do not use branches, leaves, etc., so the stock of harvested tree species should be considered. Therefore, the following calculation method is finally obtained:

(10) $$C_{STEM,j,t}=V_{TREE,j,t}*WD_{j}*CF_{j}*\frac{44}{12}$$

If the whole tree is cut down, it is the aboveground biomass carbon reserves and can be calculated in the way described above in (1). In addition, during the processing of wood, a certain loss will be generated and the loss of different tree species also varies greatly, specifically in terms of the yield rate $TOR_{ty,j}$ and the waste rate $WW_{ty}$. In summary, as for the calculation of the carbon storage amount of wood products, this article stipulates that the carbon fixed by the logs actually consumed during the service life of wood products serves as the carbon sequestration stock of wood products. The calculation formula is as follows:

(11) $$\Delta C_{HWP,t}=\underset{ty=1}{\sum }\;\underset{j=1}{\sum }\left[\left(C_{STEM,j,t}*TOR_{ty,j}\right)*\left(1-WW_{ty}\right)*OF_{ty}\right]$$

#### 2.4.2. Prediction Model of Forest Stock Volume

Trees will grow with age but, so far, there is no specific growth model of trees. The current study of growth function is mainly based on the form of function fitting; the total process curve of tree growth changes in line with the “slow—vigorous—slow—stop” process and the growth curve is similar to the “S” shape. So, theoretically, the biological model is more suitable to fit different tree species. However, a single model cannot fit the growth curves of all tree species due to different factors, such as environmental climate, planting density, and tree species. Therefore, generally speaking, a variety of data will be selected during the analysis, fitted separately and summarized, and, finally, the most appropriate model will be selected for calculation.

At present, the theoretical models commonly used in forestry tree fitting include: logistics model, Richards model, Korf model, Gompertz model, Mitscherlich model, etc.

The logistic model is the most commonly used model to simulate population dynamics; it was first proposed by P.F. Venshulst in 1838 and the standard formula of the model is as follows [35]:

(12) $$y=\frac{a}{1+b\cdot e^{-c\cdot A}}$$

The Richards growth function is a kind of theoretical growth model derived by the mathematical deduction method. It is the most accurate and suitable growth model for describing biological growth processes. It is improved by FJ. Richards according to the VonBertalanffy growth theory. It generally has a good fitting effect when describing the growth of a single wood or a stand. The pattern standard formula is as follows [36]:

(13) $$y=a\cdot {\left(1-e^{-b\cdot A}\right)}^{c}$$

The Korf model was first proposed by Korf in 1939. Later, forestry researchers achieved good results when describing tree height and breast diameter growth by using the Korf model. The model standard formula was as follows [37]:

(14) $$y=a\cdot e^{-b\cdot A^{-c}}$$

The Gompertz model is a model proposed by the British mathematician Gompertz B (1825) when describing the human age distribution, death curve, and other factors [38]. Wright (1926) used this model as a growth equation to describe growth. Many scholars later found that the Gompertz model is a typical “S” curve with initial values, which is more suitable for describing tree growth [39]:

(15) $$y=a\cdot e^{-b\cdot e^{-c\cdot A}}$$

The Mitscherlich model was proposed by Mitscherlich to describe the response of plant growth to environmental factors, with the following equation [40]:

(16) $$y=a\cdot \left(1-e^{-b-A}\right)$$

According to previous studies, the main statistical indicator to evaluate the effect of the regression model fitting is the standard error (SEE) of the correlation coefficient (R^2^) estimate. Different data were merged separately for analysis:

(17) $$R^{2}=1-\frac{\sum_{t=1}^{n}{\left(y_{i}-\hat{y}\right)}^{2}}{\sum_{l=1}^{n}{\left(y_{i}-\bar{y}\right)}^{2}}$$

(18) $$SSE=\overset{n}{\underset{i=1}{\sum }}{\left(y_{i}-\hat{y}\right)}^{2}$$

Here, the fitting results of the different models are presented with the results of wetland pine in the forest, as shown in Table 3.

Among all the results, the correlation coefficients of logistic, Richards, and Gompertz are the three highest and are also relatively close, but the Richards model has a huge difference in the amount of data that is not biologically significant, while the formula is complex and prone to large errors. At the same time, it is easy for the complex formula to cause large errors. After further comparing the logistic model with the Gompertz model, the other regression parameters of the Gompertz model, SEE, TRE, MSE, MPE, MPE, and MPSE, were found to be lower than the logistic model, which indicates that the Gompertz model is more highly fit. So, this paper finally used the Gompertz model as its growth model. Similarly, the growth models of other dominant tree species in the forest were compared and analyzed, and, finally, the best growth models of different tree species were obtained, as shown in Table 4.

Selection of the forest resulted in most trees in this forest conforming to the Gompertz model. However, in some small-scale tree species, the logistic model was found to better fit its growth curve. Therefore, managers need to obtain the specific optimal growth curve belonging to the forest, either by actual measurements or by consulting the literature, according to the specific conditions of the forest.

After obtaining the best growth model for each forest species, the increased stock curve of each forest species within 100 years was calculated over 100 years and then summarized to obtain the stock growth of the whole forest.

#### 2.4.3. Construct a TEV Forest Evaluation Model

The biggest benefit of forest managers is the value of carbon emission rights in the Paris Agreement, followed by the management of tourism, the management value of forest culture, and, also, the value of management resources, such as forest land value and forest tree value. At present, the mainstream evaluation method of forest value is the TEV model proposed by American economist A. Freeman, who refers to TEV as a resource value system in a sustainable development framework. This paper combines the actual statistics of the forestry field, and the comprehensive value of the forest is selected.

(1) Economic value evaluation of forest land resources

Selecting the woodland, other shrubs, and sparse woodland, a certain slope and its corresponding area is measured and the annuity capitalization method is used to calculate the price of the reference land.

Calculated using the average annual rental income price of arable land in the vicinity of this forest land resource, the average land rental yield assessment method is used and the local cultivated land rental price is USD 417 per hectare. In 2017, one-year treasury bonds had an average yield rate of 2.6% as the benchmark interest rate, with land rent for 20% of the pure income, referring to the land price of USD 324 per hectare. *V* represents the economic value of forest land resources, $A_{i}$ is the average rent of forest land in year *i*, and *P* represents the return on investment.

(19) $$V=\overset{n}{\underset{i=1}{\sum }}\frac{A_{i}}{P}$$

(2) Evaluation of the economic value of forest tree resources

In this study, the direct market approach was used to assess the amount of forest resource asset value. It is assumed that all forest trees can be cut, and their log value amounts are calculated based on the stocking volume of each tree species to represent the forest resource value amount. Through the land area of various forest trees, the log price, and the average log output rate, the economic value of the forest tree resource assets is evaluated by adopting the market value method.

(3) Value assessment of carbon fixation

In the calculation process, the value of carbon sequestration function is mainly accounted for by the carbon emission trading price, and the value of carbon sequestration function is accounted for by the net production power of plants and the carbon sink trading price. $F_{s}$ represents the value of carbon fixation, B represents the net plant productivity, A represents the forest area, and $P_{a}$ represents the carbon emission trading price, which comes from the record information table of the carbon GSP project in Wengyuan County released by the Guangdong Provincial Department of Ecology and Environment in Shaoguan City. The formula is as follows:

(20) $$F_{s}=1.63*0.2727*B*A*P_{a}$$

(4) Assessment of biological habitat value

According to existing studies, deforestation causes organisms to lack sufficient habitat to reproduce [41], and the loss is USD 400/ha, so the actual willingness to pay for the forest ecology will reach USD 112/ha. Based on these data, biological habitat values can be calculated.

(21) $$M_{a}=T_{a}*\left(400+112\right)*A_{c}$$

$M_{a}$ represents the value of the biological habitat, $T_{a}$ represents the forest area, and $A_{c}$ represents the exchange rate.

## 3. Results and Discussion

### 3.1. Quantifying the Amount of Carbon Dioxide Sealed by Forests and Their Products

In order to show the carbon reserve data more intuitively, four forests were selected as samples for calculation. Table 5 shows the area of the tree species and the calculated average carbon fixation per mu.

As can be seen from the table, the different forest farms plant different tree species, different forest area makes the forest average per mu of carbon, and more eucalyptus, soft broad and hard broad species are planted in State Business Forest Farm, which makes the carbon sequestration efficiency of this forest site higher than the other three forest fields; Comparing the four forest data, it can be concluded: the expansion of the total forest area is a direct factor for the expansion of forest carbon sequestration, while the change in forest species and the area of different forest species are also important factors affecting the forest carbon sequestration efficiency.

### 3.2. Forest Prediction Results

After 100 years of iteration, the stock growth volume per unit area of various tree species was obtained within 100 years, as shown in Table 6.

The average annual increase in storage per unit area over 100 years was multiplied by the unit area and time of each forest species to predict the increase in storage. The results showed that the predicted growth of forest stock after 100 years in Longxian Town was 29,465.7 m^3^; it is predicted that the growth amount of forest stock in Xinjiang Town forest farm after 100 years is 11,887.7 m^3^. It is predicted that the growth amount of forest stock after 100 years is 13,495.6 m^3^. It is predicted that the growth amount of forest stock in Yuanfeng Forest Farm after 100 years is 14,596.2 m^3^. Comparing the existing stock in the forest, it was found that the stock of soft broad and hard broad species grew faster, while the stock of mixed conifer and fir grew slower.

### 3.3. Forest Value Evaluation Results

In this study, the four forest farms located in Guangdong Province, China, were selected, and the data of the four forest farms were imported into the TEV model (see Table 7).

The data obtained from the TEV model for each value show that, in the composition system of the total value of the four forest sites, the forest land value and the forest tree value are much higher than the carbon sequestration value and the biological habitat value of the forest sites, so the assets brought by the forest farm are far greater than the assets brought to the forest farm by maintaining the original state. Because the forest farm is located in southern China and the value of the tree species planted is relatively high, the value of the forest trees in the four forest farms is far greater than the ecological value of the forest farm.

### 3.4. Discussion

By comparing the forest area and carbon storage of the four forest farms, it can be seen that, even with a wider forest area and a large number of tree species advantageous for carbon sequestration, carbon sequestration efficiency will be reduced if large numbers of trees are harvested. If kept in a reasonable cutting range and constantly planting saplings, the carbon fixation efficiency of forests will increase significantly. The results of the study by Stephen R et al. demonstrate that the times required for bioenergy substitutions to repay the C debt incurred from biomass harvest are usually much shorter (<100 years) than the time required for bioenergy production to substitute the amount of C that would be stored if the forest were left unharvested entirely [42]. It has been shown that higher carbon prices increase the optimal harvesting age [43]. These studies are consistent with our results. In addition, when the rotation length increases, the forest’s tree carbon stock increases [44].

When predicting the carbon storage of the forest 100 years later, it is found that, when the management plan cycle increases to 10 years, the most intuitive impact is the carbon sequestration efficiency of wood products. Because the related function of wood products is an exponential function, the attenuation speed is asynchronous, that is, the making of building materials from wood products has a better effect on carbon retention. At the same time, considering the growth cycle of trees, the improvement efficiency of accelerated forest wood products in 10 years is far lower than that of rosewood and other trees that need to grow for a long time. Therefore, when the management plan cycle increases by 10 years, it is necessary to change the tree varieties planted in the forest with a rare but slow growth cycle. On the one hand, its relatively small tree age is more conducive to the growth of stock and, on the other hand, it can produce higher economic value and maximize the total income. Studies show that mature forests grow more slowly than young forests [45]. Others studies have similarly shown that younger stands are preferred at the expense of old-growth stands with the carbon index [46].

Finally, the overall value of the forest is assessed and the value of the forest trees accounts for the majority of the total value; the value of forest trees is mainly reflected in forest tree products. The retention rate of carbon in different wood products is not necessary. Making wood into buildings, furniture, and saw materials and other related products can effectively maintain the products and improve the amount of carbon retention. So, to improve the efficiency of carbon sequestration in forests, more buildings, furniture, and saw materials should be made, while the manufacturing of packaging materials, cardboard, and salary materials should be reduced. Some results also indicate that forest carbon sequestration is a low-cost abatement method [47,48,49]. In addition, wood products are considered to contribute to the mitigation of carbon dioxide emissions [50].

## 4. Conclusions

In order to alleviate the global warming caused by greenhouse gas emissions and improve the carbon fixation efficiency and overall value of the forest, this paper, on the basis of the biomass conversion factor method, enriches the way of calculating forest carbon reserves, refines the precision of calculating forest carbon reserves based on the biomass conversion factor method, and proposes the method of TEV value evaluation according to the specific forest farm data. Forest farm managers cannot simply expand the area of forest farm and should realize that the choice of forest species is the best way to improve carbon fixation efficiency. In addition, the value of forest land and forest value can be used as the management direction of forest managers. The proposed area is the forest located in the middle subtropical monsoon climate and dominated by herbaceous vegetation and mixed coniferous and broad-leaved forests; the specific recommendations are as follows: first, plant more varieties of broad-leaved trees. This paper selected some representative tree varieties and found that the broad-leaved tree varieties had the highest efficiency of carbon fixation at the same stock volume. This is similar to the actual situation, where broadleaf trees have a larger leaf area compared to other trees and can also continue to accumulate biomass during the winter. Therefore, managers can appropriately increase the number of broad-leaved tree varieties according to the actual situation. Second, grow more younger trees. The results showed that tree chest diameter (DBH) and tree height (H) affected tree biomass, thus affecting the efficiency of carbon retention, and the rate of tree growth curve decreased with increasing age. The younger the tree species in the same tree, the more efficient it is at sequestering carbon. Therefore, forest managers can plant younger trees in the forest to effectively improve the overall carbon sequestration efficiency of the forest. Third, increase the vertical distribution pattern of forests. In addition to increasing carbon sequestration in forests, shrubs can also increase carbon sequestration. If there are too many forest shrubs, shrubs can crowd out sunlight, nutrients, and space for seedling growth in order to hinder seedling development, thus reducing the effectiveness of forest-type carbon sequestration. Therefore, in order to increase the carbon sequestration of forests, it is necessary to appropriately increase the number of shrubs in the forest and ensure that they do not affect the growth of trees. Fifth, make the trees into a longer retention time of wood products. The most important source of income of the forest farm is the economic income produced by logging and making wood products. According to the policy requirements and the forest growth cycle, the forest tree resources should be made into wood products with a longer retention time, so as to improve the total value of the forest.

## Figures and Tables

**Table 1:** Reference Values.

Region	$DF_{DW}$	Region	$DF_{DW}$
northeast	3.51%	Central China	2.25%
NC	2.06%	south China	2.25%
Inner Mongolia	3.51%	southwest	1.88%
Central Plains	2.06%	northwest	3.11%

**Table 2:** Reference values of the parameters a and b.

Varieties of Trees	Parameter a	Parameter b
Spruce, fir	20.7385	−0.0102
Dahurian larch	67.413	−0.0141
Chinese pine	24.8265	−0.0234
Chinese red pine	7.21751	−0.0067
Other pine species (including Simao pine, Yunnan pine, white pine, torch pine, red pine, camphor pine, Huashan pine, wetland pine, etc.)	13.1198	−0.009
Cedarwood	3.75954	−0.0047
Cedar trees and other Chinese fir species	4.98967	−0.0025
Quercus	7.73245	−0.0048
Other hard broad class (birch, maple, lotus, water, yellow, camphor tree, nanmu, etc.)	6.9779	−0.0043
poplar	12.3106	−0.0069
eucalyptus	24.6966	−0.0137
yearning between lovers	9.53883	−0.0004
Other soft broad (polar, wood, willow, aulownia, nanmu, casuarhedra, etc.)	8.12855	−0.0046

**Table 3:** Model fitting results.

Model	Fits the Equation	Correlation	The Sum of the Squares of the Residue
Logistic	$y=\frac{0.168}{1+217.754\cdot e^{-0.166\cdot x}}$	0.8067	0.0205
Richards	$y=4647673.5\cdot {\left(1-e^{-6.7E-5\cdot x}\right)}^{2.965}$	0.7632	0.0314
Gompertz	$y=1.54\cdot e^{-8.434\cdot e^{-0.034\cdot x}}$	0.7592	0.0312
Korf	$y=4758635.5\cdot e^{-30.758\cdot x^{-0.157}}$	0.7463	0.0320
Mitscherlich	$y=31568.9\cdot \left(1-e^{-3.6E-8\cdot x}\right)$	0.6157	0.0666

**Table 4:** Best growth models for the predominant tree species.

Dominant Tree Species	Optimal Growth Model
eucalyptus	$V=2.81\cdot e^{-6.434\cdot e^{-0.124\cdot t}}$
Chinese red pine	$V=3.437\cdot e^{-8.14\cdot e^{-0.102\cdot t}}$
Soft broad class	$V=3.69\cdot e^{-7.328\cdot e^{-0.164\cdot t}}$
China fir	$V=1.36\cdot e^{-8.724\cdot e^{-0.104\cdot t}}$
Hard broad class	$V=3.86\cdot e^{-7.414\cdot e^{-0.134\cdot t}}$
Needle wide mix	$V=1.63\cdot e^{-3.534\cdot e^{-0.184\cdot t}}$
Needle leaf mix	$V=1.54\cdot e^{-8.434\cdot e^{-0.034\cdot t}}$
Castanopsis fissa	$V=2.364\cdot e^{-7.431\cdot e^{-0.147\cdot t}}$

**Table 5:** Tree species area and carbon reserves of the forest farm.

	Tree	Longxi Town Forest Farm	Xinjiang Town Forest Farm	State Business Forest Farm	Source Feng Forest Farm
Accumulation (m^3^)					
eucalyptus		9836	25,441	107,098	57,896
Chinese red pine		21,228	15,463	5629	681
Soft broad class		43,502	0	15,248	15,248
China fir		10,096	30,960	442	442
Hard broad class		0	0	9343	9343
Needle wide mix		12,089	0	1492	0
Needle leaf mix		863	0	0	1492
Castanopsis fissa		0	0	1073	0
Total forest area (h a)		2074.5	1653.9	2643.9	20,386
Average carbon sequestration per acre (t CO_2_-e ha^−1^)		75.64	65.12	85.63	70.39
Forest carbon reserves (t CO_2_-e)		156,924	107,701	226,388	14,347

**Table 6:** Increased stock volume over 100 years per unit area of the dominant tree species.

Dominant Tree Species	Average Annual Stock Increase Per Unit Area over 100 Years (m^3^)
eucalyptus	2.2713
Chinese red pine	2.5532
Soft broad class	3.1304
China fir	1.0080
Hard broad class	3.1359
Needle wide mix	1.4743
Needle leaf mix	0.4376
Castanopsis fissa	1.9604

**Table 7:** Results of comprehensive economic value evaluation of forest farm.

Value Category (CNY 10,000)	Longxi Town Forest Farm	Xinjiang Town Forest Farm	State Business Forest Farm	Source Feng Forest Farm
Value of forest land resources	429.83	392.31	627.13	483.56
Tree value	4914.14	3969.97	5798.75	3727.58
Carbon fixation value	59.41	67.48	18.63	44.87
Biological habitat value	15.93	12.70	20.30	15.66

## Data Availability

Not applicable.
